# Immune-related genes in tumor-specific CD4^+^ and CD8^+^ T cells in colon cancer

**DOI:** 10.1186/s12885-020-07075-x

**Published:** 2020-06-22

**Authors:** Xi Yang, Wei Wu, Yuefen Pan, Qing Zhou, Jiamin Xu, Shuwen Han

**Affiliations:** 1 Department of Oncology, Huzhou Cent Hosp, Affiliated Cent Hops HuZhou University, No. 198 Hongqi Road, Huzhou, 313000 Zhejiang Province China; 2 Department of Gastroenterology, Huzhou Cent Hosp, Affiliated Cent Hops HuZhou University, No.198 Hongqi Road, Huzhou, 313000 Zhejiang Province China; 3 Department of Critical Care Medicine, Huzhou Cent Hosp, Affiliated Cent Hops HuZhou University, No. 198 Hongqi Road, Huzhou, 313000 Zhejiang Province China; 4 Graduate School of Nursing, Huzhou University, No. 1 Bachelor Road, Huzhou, 313000 Zhejiang Province China; 5 Department of Oncology, Huzhou Cent Hosp, Affiliated Cent Hops HuZhou University, No. 198 Hongqi Road, Huzhou, 313000 Zhejiang Province China

**Keywords:** Colon cancer, Immunity, Competing endogenous RNAs, CD4^+^ T cells, CD8^+^ T cells

## Abstract

**Background:**

Immune escape is an immunological mechanism underlying tumorigenesis, and T cells play an important role in this process. In this study, immune-related genes were evaluated in tumor-infiltrating CD4^+^ and CD8^+^ T cells in colon cancer.

**Methods:**

ESTIMATE was used to calculate stromal and immune scores for tumor datasets downloaded from The Cancer Genome Atlas–Colon Cancer (COAD). Differentially expressed genes (DEGs) between samples with high and low stromal and immune scores were screened, followed by a functional enrichment analysis of the overlapping DEGs. The DEGs related to CD4^+^ and the CD8^+^ T cells were then screened. Predicted miRNA–mRNA and lncRNA–miRNA pairs were used to construct a competing endogenous RNA (ceRNA) network. Furthermore, chemical–gene interactions were predicted for genes in the ceRNA network. Kaplan–Meier survival curves were also plotted.

**Results:**

In total, 83 stromal-related DEGs (5 up-regulated and 78 down-regulated) and 1270 immune-related DEGs (807 up-regulated and 293 down-regulated genes) were detected. The 79 overlapping DEGs were enriched for 39 biological process terms. Furthermore, 79 CD4^+^ T cell-related genes and 8 CD8^+^ T cell-related genes, such as ELK3, were screened. Additionally, ADAD1 and DLG3, related to CD4^+^ T cells, were significantly associated with the prognosis of patients with colon cancer. The chr22-38_28785274–29,006,793.1–miR-106a-5p-DDHD1 and chr22-38_28785274–29,006,793.1–miR-4319-GRHL1 axes obtained from CD4^+^ and CD8^+^ T cell-related ceRNAs were identified as candidates for further studies.

**Conclusion:**

ELK3 is a candidate immune-related gene in colon cancer. The chr22-38_28785274–29,006,793.1–miR-106a-5p-DDHD1 and chr22-38_28785274–29,006,793.1–miR-4319-GRHL1 axes may be related to CD4^+^ and CD8^+^ T cell infiltration in colon cancer.

## Highlights

1. ELK3 is a candidate immune-related gene in colon cancer
2. The chr22-38_28785274–29,006,793.1–miR-106a-5p–DDHD1 axis was identified in an analysis of CD4^+^ T cell-related ceRNA networks
3. The chr22-38_28785274–29,006,793.1–miR-4319–GRHL1 axis was identified in an analysis of CD8^+^ T cell-related ceRNA networks
4. The CD4^+^ T cell-related genes ADAD1 and DLG3 were associated with prognosis
5. A total of 175 chemical–target pairs in CD4^+^ T cells and 9 in CD8^+^ T cells were obtained

## Background

Colon cancer is among the worst cancers with respect to mortality and incidence worldwide, especially in Asia [1]. Despite considerable advances in surgical and adjuvant therapy for colon cancer, rates of recurrence for patients with stages I–III and stage IV cancer are 30 and 65%, respectively [2]. Various issues, including resistance, relapse, and metastasis occurring after traditional radiotherapy, chemotherapy, and new targeted drug treatments, have revealed that tumors are a systemic disease and not simply a result of mutations in oncogenes and the inactivation of tumor suppressor genes [3, 4]. Tumor escape from immune surveillance mechanisms has prompted the advent of tumor immunotherapy [5, 6]. Immune evasion is one of the most important characteristics of tumors [7]. It mainly occurs by the modification of tumor cells and changes in the tumor microenvironment. As such, an understanding of the mechanism underlying tumor immune escape can provide new strategies for immunotherapy [8]. The T cell-mediated immune response against tumors is the basis for cancer immunotherapy and is correlated with favorable disease outcomes [9, 10].

Tumor immune escape is related to a decline in T cell responses, mainly manifested by immune tolerance to CD4^+^ T cells and the inhibition of CD8^+^ T cell activation [11]. CD4^+^ T helper cells can assist in the activation of naive CD8^+^ T cells and can help to eliminate major histocompatibility antigen class II (MHC-II)-negative tumor cells. However, tumor cells can induce specific immune tolerance in CD4^+^ T cells [12]. During the antitumor immune response, CD8^+^ T cells play a major role in directly killing tumor cells by recognizing tumor antigens. However, the local tumor microenvironment contains a large number of cytokines that individually or synergistically affect the activation of cytotoxic T lymphocytes (CTL) and the sensitivity of tumor cells to CTL activity [13]. Via interactions among various immune elements, cancer cells may enter a dormant state or exhibit immune evasion, which may directly promote tumor development and progression. Therefore, we analyzed the molecular mechanisms associated with CD4^+^ and CD8^+^ T cell activity in colon cancer to explore the potential benefits of immunotherapy.

To provide a basis for the investigation of colon cancer-induced T cell-mediated immune escape, miRNA target prediction and competing endogenous RNA (ceRNA) network construction were performed. Furthermore, prognostic targets were evaluated, and drug screening was performed to screen prognostic indicators and strategies for colon cancer immunotherapy, which may provide guidance for clinical decision-making. In our study, datasets for colon cancer samples were downloaded from The Cancer Genome Atlas (TCGA) public database. Moreover, we performed an enrichment analysis of the differentially expressed genes (DEGs) correlated with both stromal and immune scores. CD4^+^ and CD8^+^ T cell-related DEGs were obtained and used for analyses of protein–protein interactions (PPI). Additionally, the ceRNA networks for CD4^+^ and CD8^+^ T cells were analyzed and small chemical molecules related to the DEGs in the ceRNA network were predicted. Finally, a survival analysis of the CD4^+^ and CD8^+^ T cell-related DEGs was performed.

## Methods

### Data source

**Fig. 1:**
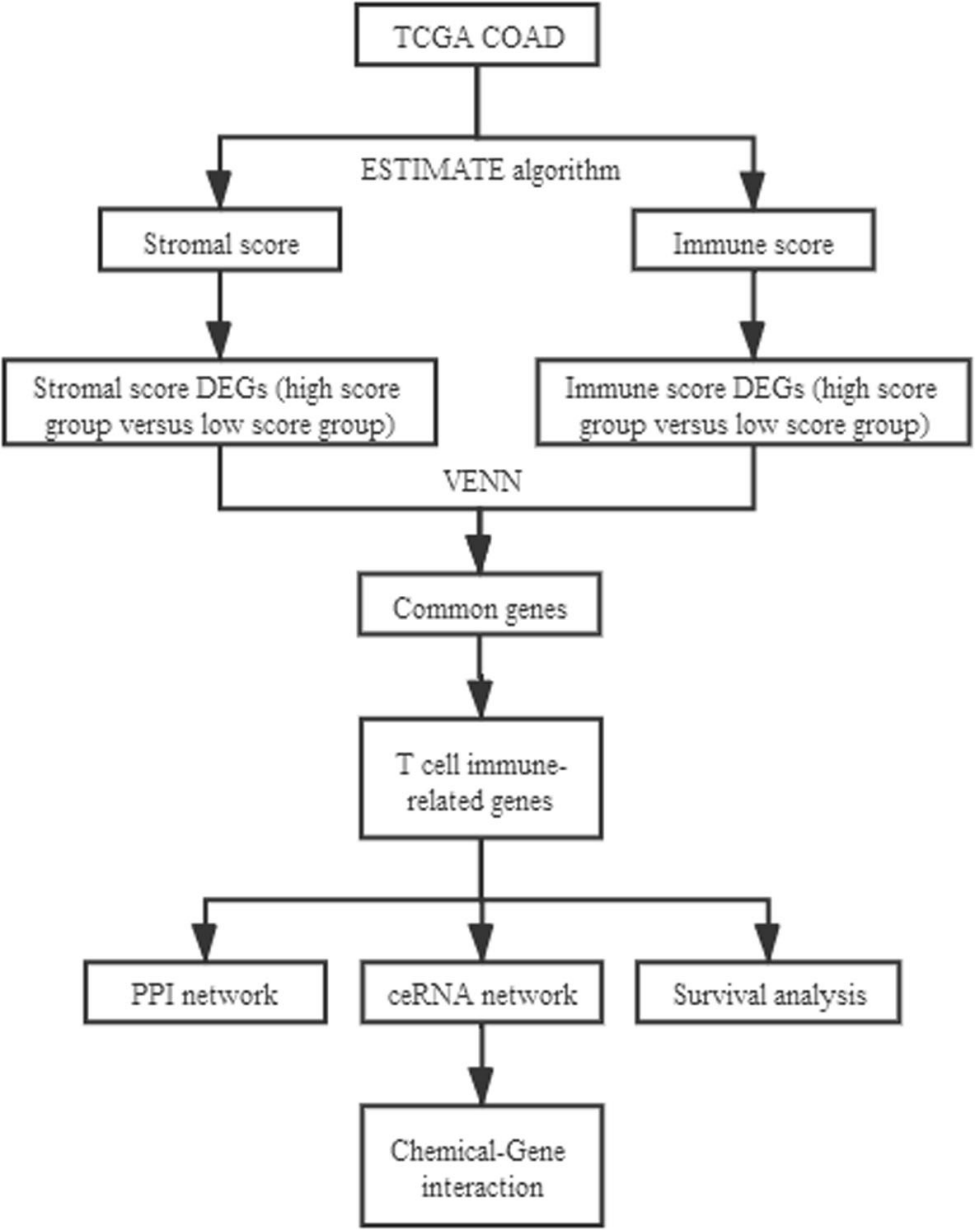
Workflow of dataset processing

The GDC TCGA Colon Cancer (COAD) dataset (version 07-19-2019) was downloaded from TCGA (https://xenabrowser.net/), including RNAseq FPKM data and corresponding clinical phenotypes. The dataset included data for 448 colon cancer samples. The data were analyzed according to the workflow illustrated in Fig. 1.

### Analysis of stromal and immune scores

For the mRNA expression profile analysis, gene expression values were calculated by mapping probes (obtained from the microarray dataset and the annotation files for the chip platform) to gene symbols. The average mRNA expression level was obtained when multiple probes matched to one symbol.

To analyze stromal and immune cell infiltration in tumor tissues, Estimation of Stromal and Immune cells in Malignant Tumours using Expression data (ESTIMATE, version 1.0.13) [14] in R was used to predict tumor purity. Thus, the stromal and immune scores for each tumor sample were obtained.

### wAnalysis of differentially expressed genes

Based on the median stromal score, the tumor samples were divided into groups with high and stromal scores. Likewise, the tumor samples were divided into groups with high and low immune scores according to the median immune score.

The typical Bayesian test in the limma package (Version 3.10.3) [15] was used to analyze differentially expressed mRNAs (dif-mRNAs) between the two stromal score groups and two immune score groups. mRNAs with a fold change value of more than 0.263 and a *p*-value of less than 0.05 were selected as dif-mRNAs.

To identify potential regulatory genes associated with both stromal and immune cell contents, the overlapping DEGs were selected and represented by a Venn diagram.

### Enrichment analysis

Utilizing the clusterProfiler tool [16] in R (Version 3.2.11), the co-expressed DEGs were evaluated by a Kyoto Encyclopedia of Genes and Genomes (KEGG) [17] pathway enrichment analysis and Gene Ontology [18] Biological Process (BP) enrichment analysis. The significantly enriched terms with a p-value of less than 0.05 and involving no less than two DEGs were selected.

### Screening CD4^+^ and CD8^+^ T cell-related DEGs

Based on the DEG data for the RNA-seq expression profiles, the abundance of infiltrating immune cells in tumor samples was estimated using the Cibersort algorithm [19]. The infiltration of six types of immune cells (CD4^+^ T cells, CD8^+^ T cells, B cells, neutrophils, dendritic cells, and lymphocytes) in tumor tissues was evaluated.

The Pearson correlation coefficients for the relationships between the expression levels of DEGs and the abundance of infiltrated CD4^+^ and CD8^+^ T cells were calculated. The immune-related DEGs with an absolute value of r > 0.15 were selected.

### PPI network construction

Interactions between proteins encoded by immune-related DEGs in CD4^+^ T cells were retrieved from the STRING database [20] (version 11.0) with a PPI score setting of 0.15 (low confidence) and the species set to human. Based on the retrieved PPIs, the CD4^+^ T cell-related PPI network was visualized using Cytoscape [21] (version 3.2.0). The CD8^+^ T cell-related PPI network was constructed following the same method.

### ceRNA network construction

miRWalk 3.0 [22] was used to predict target–miRNA regulatory relationships. The miRNAs correlated with DEGs related to CD4^+^ T cells were predicted, and the species was set to human. The regulatory relationships with a score exceeding 0.95 appearing in both the TargetScan and miRDB databases were selected. The miRNAs correlated with DEGs related to CD8^+^ T cells were predicted using the same method.

The lncRNA–miRNA relationships for CD4^+^ T cells were predicted using the DIANA-LncBase database v.2 [23]. The lncRNA–miRNA regulatory relationships with a score of 1 were selected. The lncRNA–miRNA and target–miRNA data were integrated to construct the ceRNA network of the lncRNA–miRNA–target relationships related to CD4^+^ T cells. A CD8^+^ T cell-related ceRNA network was constructed following the same method.

### Small chemical molecule–target network construction

The Comparative Toxicogenomics Database (CTD) [24] was used to search for colon cancer-related genes and small chemical molecules. Overlapping genes associated with colon cancer and belonging to the CD4^+^T cell-related ceRNA network were used to screen chemical–target pairs. A CD4^+^ T cell-related small chemical molecule–target network was constructed using Cytoscape. A CD8^+^ T cell-related small chemical molecule–target network was also constructed using the same method.

### Survival analysis

Clinical phenotype data related to prognosis in TCGA were collected, including overall survival (OS). The CD4^+^ T cell-related genes and were divided into high/low expression groups based on the median gene expression value and the same grouping strategy was applied to CD8^+^ T cell-related genes. Differences were evaluated by the log-rank test and genes with a *p*-value of less than 0.05 were considered significantly correlated with prognosis. Furthermore, Kaplan–Meier (K-M) survival curves were plotted.

## Results

### Differences in gene expression between colon cancer with high and low stromal and immune scores

**Fig. 2:**
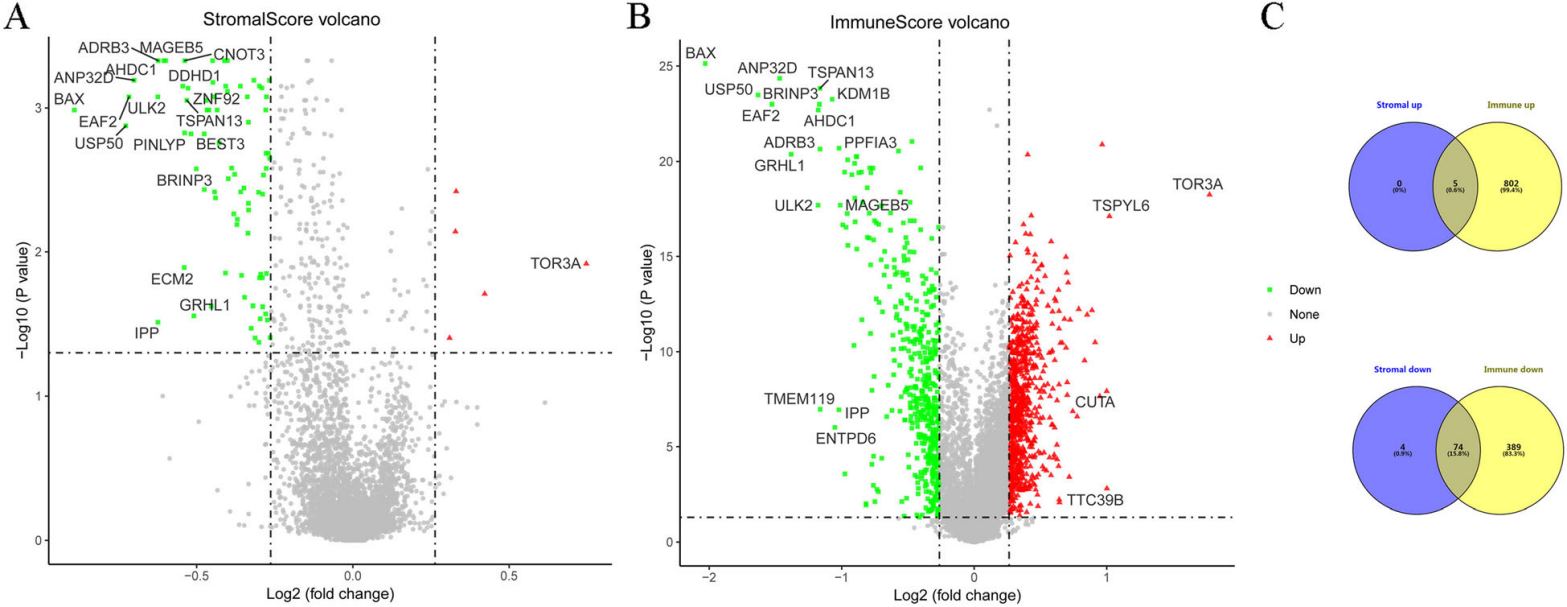
Gene expression profiles for colon cancer tissue samples. Volcano plot showing the expression profiles of genes in groups with high or low stromal scores (**a**) and with high or low immune scores (**b**). The red and green dots in the volcano plot represent up-regulated and down-regulated genes, respectively. Venn diagram (**c**) showing overlapping differentially expressed genes in analyses of genes related to stromal scores and immune scores. The GDC TCGA Colon Cancer (COAD) dataset (version 07-19-2019) was downloaded from TCGA (https://xenabrowser.net/). For the analysis of the infiltrating stromal and immune cells in tumor tissues, Estimation of STromal and Immune cells in MAlignant Tumours using Expression data (ESTIMATE, version 1.0.13) implemented in R was used

There were 83 DEGs (5 up-regulated and 78 down-regulated) between the groups with high and low stromal scores. A total of 1270 DEGs (including 807 up-regulated and 463 down-regulated genes) were detected between the groups with high and low immune scores. A volcano map of DEGs is shown in Fig. 2. Furthermore, 5 up-regulated and 74 down-regulated DEGs were identified in both the stromal and immune groups, as shown in a Venn diagram in Fig. 2c and in Supplementary Table 1.

### Enrichment analysis of the overlapping DEGs

**Fig. 3:**
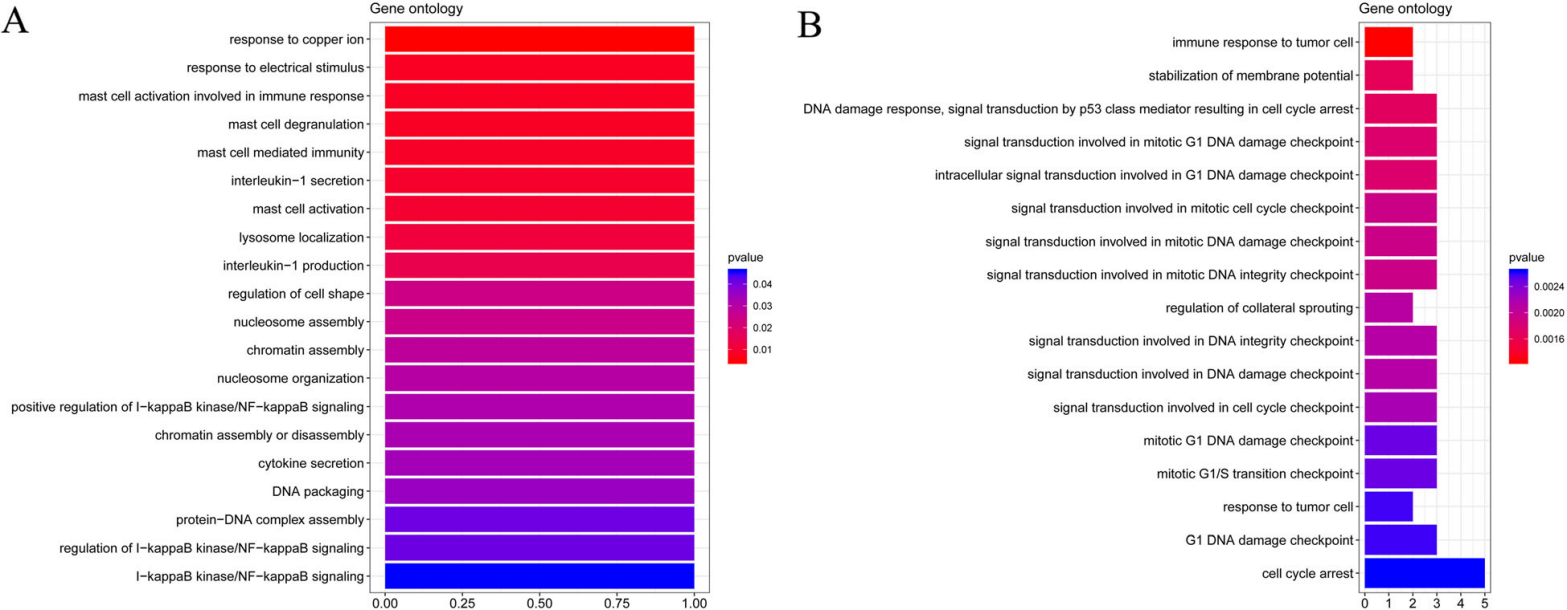
Enrichment analysis of common differentially expressed genes. Gene ontology biological process term enrichment analysis of up-regulated genes (**a**) and down-regulated genes (**b**)

In an enrichment analysis of co-expressed DEGs, up-regulated DEGs were enriched for 22 BP terms, including response to copper ions and response to electrical stimuli (Fig. 3a). The down-regulated DEGs were enriched for 17 BP terms, including immune response to tumor cells and stabilization of the membrane potential (Fig. 3b). The co-expressed DEGs were not enriched for any of the investigated KEGG pathways.

### CD4^+^ and CD8^+^ T cell-related DEGs

**Fig. 4:**
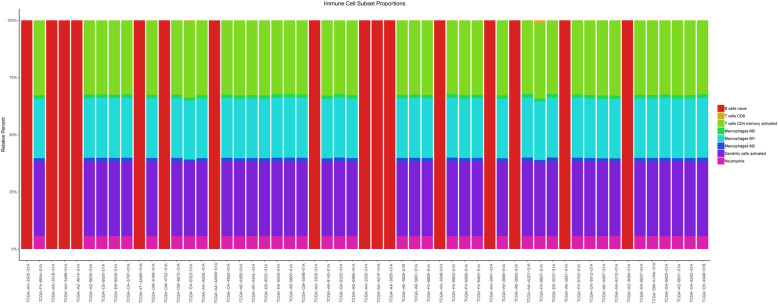
Infiltration of immune cells in colon cancer. Bar charts indicate the infiltration abundance of different immune cells. The abscissa axis represents the sample name. The longitudinal axis represents the relative percent of different types of infiltrating immune cells. Different colors indicate different types of infiltrating immune cells (B cells, CD4^+^ T cells, CD8^+^ T cells, Neutrphils, Macrophages and Dendritic cells)

Based on the Cibersort algorithm, the abundances of infiltrating immune cells (CD4^+^ T cells, CD8^+^ T cells, B cells, neutrophils, dendritic cells, and lymphocytes) in the tumor samples were estimated (Fig. 4). Based on Pearson correlation coefficients, 79 CD4^+^ T cell-related DEGs and 8 CD8^+^ T cell-related DEGs were identified.

### PPI networks of CD4^+^ and CD8^+^ T cell-related DEGs

**Fig. 5:**
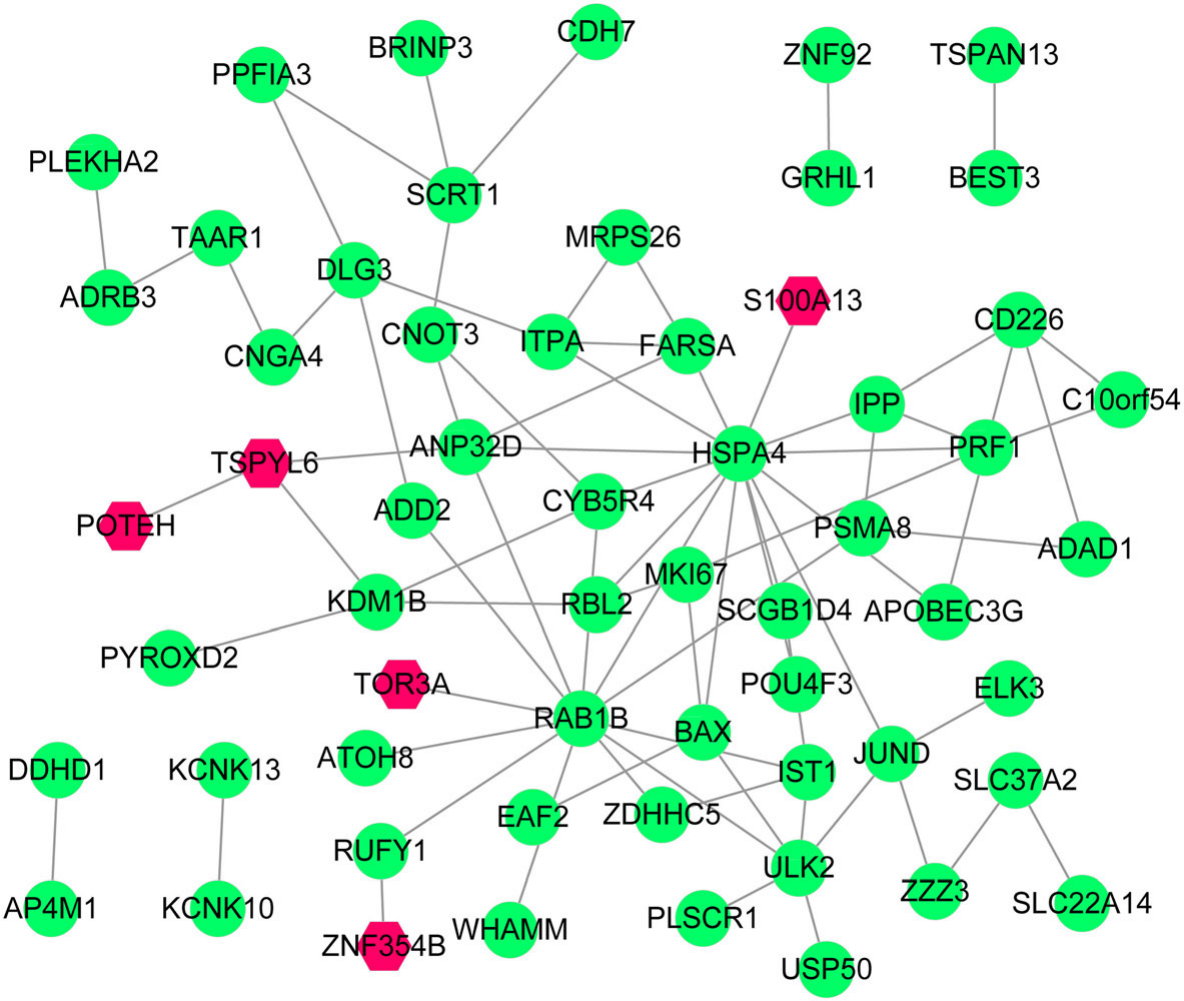
Protein–protein interaction networks. Protein–protein interaction network of CD4^+^ T cell-related genes. Red hexagons represent up-regulated genes and green nodes represent down-regulated genes

We generated a PPI network with 59 nodes and 77 interacting pairs for CD4^+^ T cell-related genes, including the ETS transcription factor ELK3, as shown in Fig. 5. There were no PPIs involving DEGs related to CD8^+^ T cells under the threshold used in this study.

### ceRNA network of CD4^+^ and CD8^+^ T cell-related DEGs

**Fig. 6:**
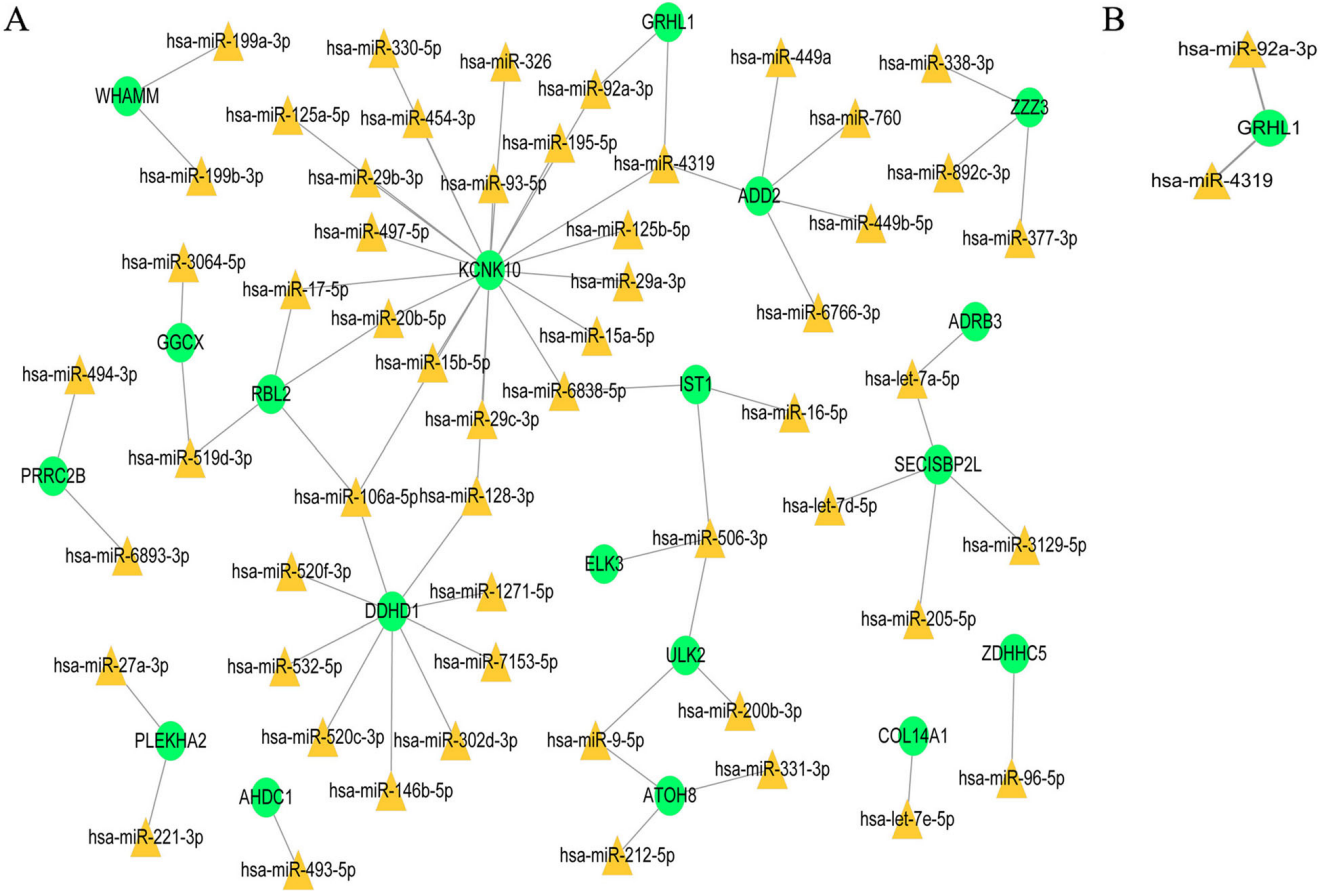
MiRNA–target regulatory networks. MiRNA–target regulatory networks of CD4^+^ T cell-related genes (**a**) and CD8^+^ T cell-related genes (**b**). Green nodes represent down-regulated genes. Yellow triangles represent microRNAs

We obtained 69 miRNA–mRNA relationships (55 miRNAs and 19 target genes) in an analysis of CD4^+^ T cells (Fig. 6a) and only two miRNA–mRNA relationships in an analysis CD8^+^ T cells, including two miRNAs and one target gene (Fig. 6b).

**Fig. 7:**
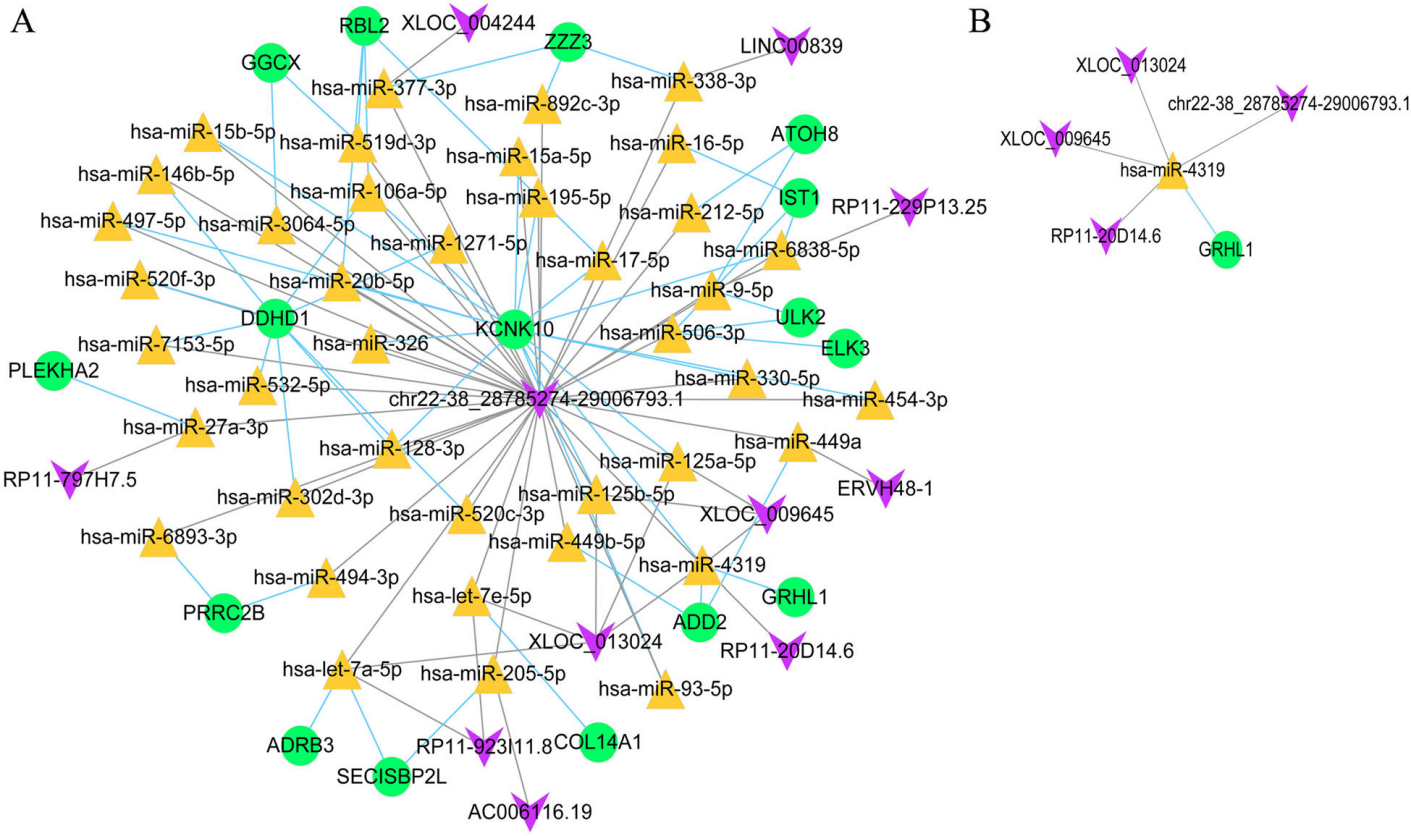
Competing endogenous RNAs (ceRNA) networks. eRNA networks of CD4^+^ T cell-related genes (**a**) and CD8^+^ T cell-related genes (**b**). Green nodes represent down-regulated genes. Yellow triangles represent microRNAs. Purple inverted triangles represent long non-coding RNAs

In total, 11 lncRNAs were predicted to interact with miRNAs in CD4^+^ T cells. After integrating these data and data for miRNA–target relationships, 110 ceRNA regulatory relationships were obtained for CD4^+^ T cells, such as chr22-38_28785274–29,006,793.1–miR-106a-5p–DDHD1, which included 45 miRNAs and 11 mRNAs (Fig. 7a).

Four predicted lncRNAs were obtained for CD8^+^ T cells. After integration with the miRNA–target relationships, five ceRNA regulatory relationships were obtained for CD8^+^ T cells, such as chr22-38_28785274–29,006,793.1–miR-4319–GRHL1, which included one miRNA and one mRNA (Fig. 7b).

### Colon cancer-related small chemical molecules target mRNAs in the ceRNA network

**Fig. 8:**
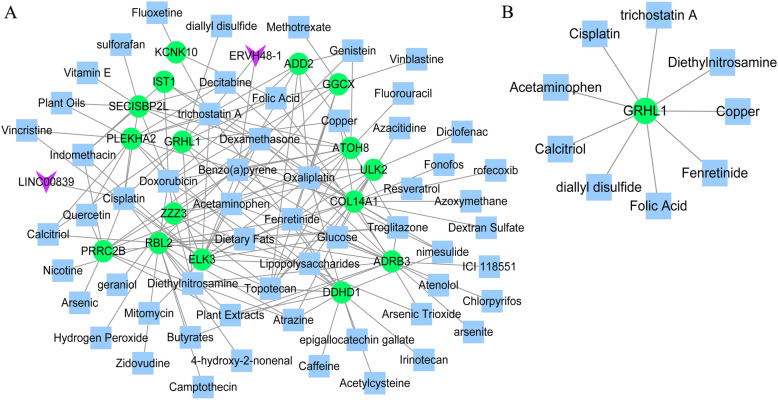
Chemical–gene interaction networks. Chemical–gene interactions were predicted for the genes in the competing endogenous RNA (ceRNA) networks by using the CTD database. The chemical–gene interaction network of genes in the CD4^+^ T cell-related ceRNA network (**a**) and CD8^+^ T cell-related ceRNA network (**b**). Green nodes represent down-regulated genes. Blue squares represent small chemical molecules. Purple inverted triangles represent long non-coding RNAs

For CD4^+^ T cells, 175 small chemical molecule–target interactions were obtained, including 2 lncRNAs, 10 genes, and 64 types of small chemical molecules (Fig. 8a). For CD8^+^ T cells, nine small chemical molecule–target interactions were obtained, including one gene and nine types of small chemical molecules (Fig. 8b).

CD4^+^ and CD8^+^ T cell-related genes associated with colon cancer prognosis.

**Fig. 9:**
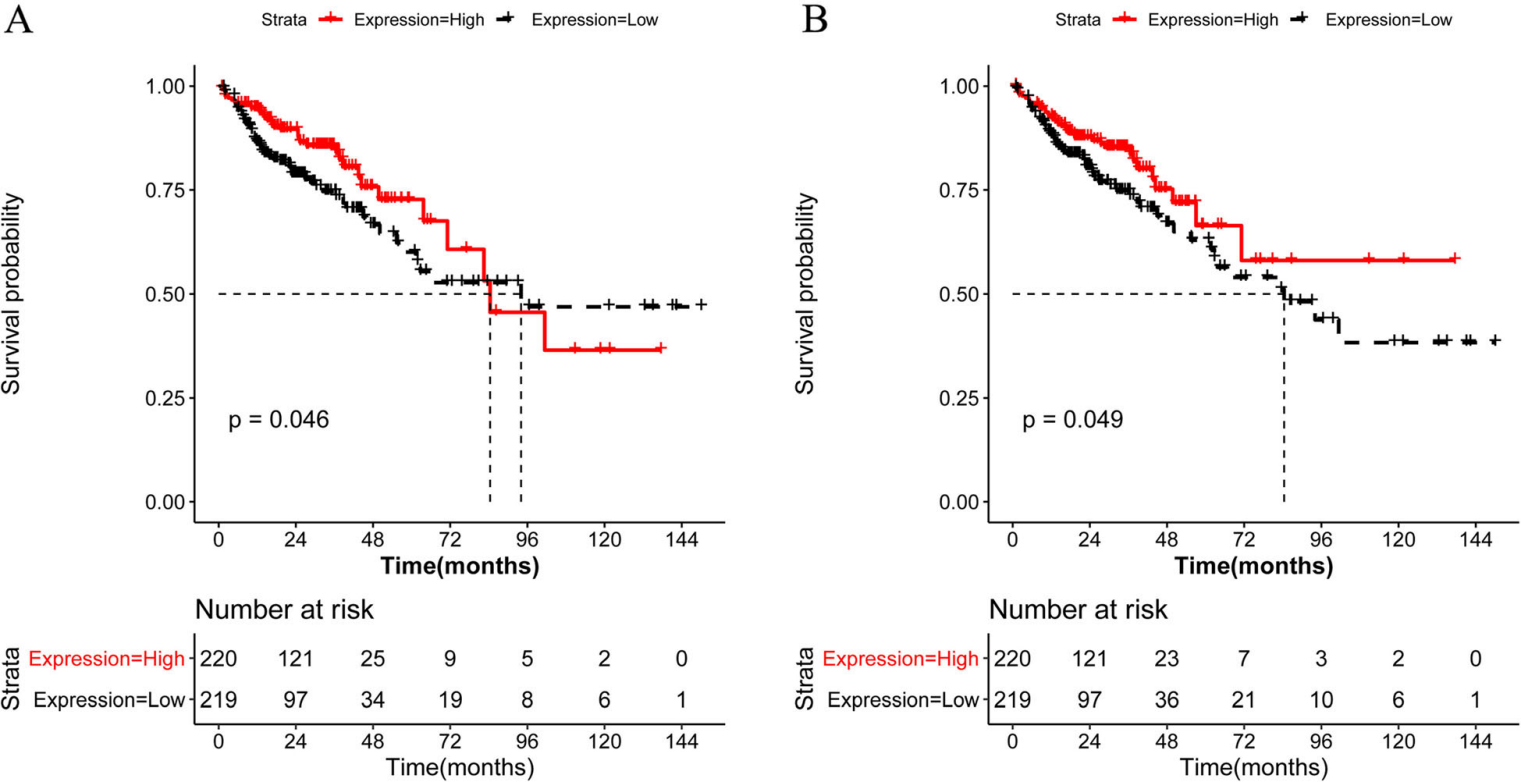
Kaplan–Meier curves of overall survival in patients with colon cancer. After screening for differentially expressed genes associated with survival, adenosine deaminase domain containing 1 (ADAD1) and the discs large MAGUK scaffold protein 3 (DLG3) were selected from 77 CD4^+^ T cell-related genes. There were no survival-related differentially expressed genes among the eight CD8^+^ T cell-related genes. Kaplan–Meier curves of overall survival showing prognosis for ADAD1 (**a**) and DLG3 (**b**)

After screening for DEGs associated with survival, adenosine deaminase domain containing 1 (ADAD1) and discs large MAGUK scaffold protein 3 (DLG3) were selected among the 77 CD4^+^ T cell-related DEGs. However, there were no survival-related DEGs among the eight CD8^+^ T cell-related genes. The K-M survival curve for ADAD1 is represented in Fig. 9a and the K-M survival curve for DLG3 is represented in Fig. 9b.

## Discussion

### ELK3 is a candidate immune-related gene in colon cancer

In this study, by comparing samples from TCGA with high or low stromal and immune scores, we obtained 83 DEGs (5 up-regulated and 78 down-regulated genes) between the two stromal score groups and 1270 DEGs (807 up-regulated and 293 down-regulated genes) between the two immune score groups. In total, 79 co-expressed DEGs were obtained (i.e., genes associated with both stromal and immune scores). The 5 up-regulated co-expressed DEGs were enriched for 22 BP terms (response to copper ions and response to electrical stimuli) and the 74 down-regulated co-expressed DEGs were enriched for 17 BP terms (immune response to tumor cells and stabilization of membrane potential). Moreover, 79 CD4^+^ T cell-related DEGs were detected, with 77 edges and 59 nodes in the PPI network, compared with only eight CD8^+^ T cell-related DEGs. Furthermore, there were 110 ceRNA relationships in the network of CD4^+^ T cell-related DEGs and five ceRNA relationships in the network of CD8^+^ T cells. Finally, to clarify the clinical applications of our findings as well as to provide a potential strategy for the prediction of clinical prognosis and targeted drug selection, prognostic genes were analyzed and small-molecule drugs were screened. In an analysis of small chemical molecule–gene interactions, 175 pairs were obtained for CD4^+^ T cells and 9 pairs for CD8^+^ T cells. In a survival analysis, ADAD1 and DLG3 in CD4^+^ T cells were most strongly correlated with prognosis. As such, analyses of the molecular mechanisms underlying the roles of CD4^+^ and CD8^+^ T cells in colon cancer may provide novel targets for immunotherapy.

ELK3 is a transcription factor belonging to the E26 transformation-specific (ETS) family [25]. The PI3K/Akt/mammalian target of rapamycin (mTOR) and ERK signaling pathways activate ELK3 [26]. Notably, studies have shown that ELK3 regulates cell migration and invasion in hepatoma cells and breast cancer [27]. In an analysis of the regulation of colorectal cancer stemness, Wang et al. demonstrated that ELK3 is involved. Moreover, ELK3 was identified as a potential target of miR-507, and its expression is restored by the abrogation of LINC00525-knockdown [28]. Many important biological processes are regulated by the ETS protein family, such as immune cell functions [29, 30]. ELK3 regulates the expression of heme oxygenase-1 (HO-1) as a transcriptional repressor. Moreover, inflammatory mediators tend to affect the expression of ELK3, which is down-regulated by bacterial endotoxins. Tsoyi et al. have shown that the ETS protein family plays a role in the immune response; during the inflammatory response to infection, ELK3 and HO-1 are important for macrophage function [31]. In our study of CD4^+^ T cell-related DEGs, ELK3 was downregulated.

### The chr22-38_28785274–29,006,793.1–miR-106a-5p-DDHD1 and chr22-38_28785274–29,006,793.1–miR-4319-GRHL1 axes may be related to CD4^+^ and CD8^+^ T cell infiltration in colon cancer

We identified the chr22-38_28785274–29,006,793.1–miR-106a-5p–DDHD1 axis from the CD4^+^ T-cell related ceRNA network as a candidate for further analyses. The phospholipase A1 (PLA1) family members are classified as extracellular and intracellular and are implicated in different intracellular mechanisms. As a phosphatidic acid (PA)-preferring PLA1 (PA-PLA1), intracellular DDHD1 has been studied extensively owing to its implications for cancer development. DDHD1 is involved in the synthesis of lysophosphatidylinositol (LPI) [32]. LPI activity is correlated with tumor growth and aggressiveness via its interaction with G protein-coupled receptor 55 (GPR-55) [33–36]. Moreover, DDHD1 supports the proliferation and survival of colon cancer cells. Studies have also demonstrated that the inhibition of the MAPK/ERK and PI3K/Akt signaling pathways reduce the viability of colon cancer cells in vitro, and apoptotic cell death is increased by the silencing of DDHD1 via small interfering RNA [37]. Our results were consistent with those of previous studies, which supported the effects of the lysophospholipid mediator DDHD1 on tumor processes. In neoplastic cells, by interacting with GPR-55, LPI induces ERK and the Akt signaling [34]. MiR-106a-5p belongs to the miR-17 family. According to the consensus seed region, there are three clusters in the miR-17 family. MiR-106a-5p is located on Xq26.2, which belongs to the miR-106a-363 cluster. MiR-106a-5p is highly expressed in gastric [38–42], breast [43, 44], colorectal [45], and non-small cell lung cancers [46]. In squamous cell carcinomas [47], colon cancers [48], and gliomas [49], miR-106a-5p is expressed at relatively low levels. Studies have also shown that in colorectal cancer, the inhibition of the anti-metastatic gene transforming growth factor-b receptor 2 (TGFBR2) increases cell migration and invasion via miR-106a-5p [45]. In our study, DDHD1 was identified as a potential target of miR-106a-5p in colon cancer cells, which influences disease progression.

In our study, the chr22-38_28785274–29,006,793.1–miR-4319–GRHL1 axis from the CD8^+^ T-cell related ceRNA network was identified as a candidate for further analyses. Grainyhead-like 1 (GRHL1) belongs to the GRHL transcription factor family, which comprises GRHL1, GRHL2, and GRHL3 [50]. Studies have suggested that the Grainyhead family genes exhibit homologous DNA-binding immunoglobulin folding to the DNA-binding domain of the key tumor suppressor p53 and that these genes participate in wound healing, embryonic neural tube closure, and epidermal integrity [51–53]. Recent studies have shown that these transcription factors are involved in various cancers, such as skin squamous cell carcinoma, gastric cancer, breast cancer, colorectal cancer, and cervical cancer [54]. Moreover, GRHL2 knockdown in colorectal cancer cells inhibits cell proliferation by targeting ZEB1 [55]. Huang et al. revealed that the expression of miR-4319 is inversely related to patient survival in colorectal cancer. Moreover, the overexpression of miR-4319 markedly reduces colorectal cancer cell proliferation by infecting ankyrin repeat and BTB domain-containing 1 (ABTB1) and alters the cell cycle distribution [56]. Thus, we hypothesized that the chr22-38_28785274–29,006,793.1–miR-4319–GRHL1 axis is correlated with CD8^+^ T cell functions and with the pathogenesis of colon cancer.

## Conclusions

A series of bioinformatics analyses were conducted to identify and characterize DEGs related to CD4^+^ and CD8^+^ T cells in colon cancer tissues. ELK3, which was down-regulated in cancer tissues, may be correlated with colon cancer and CD4^+^ T cells. Moreover, the chr22-38_28785274–29,006,793.1–miR-106a-5p–DDHD1 axis from the CD4^+^ T cell-related ceRNA network as well as the chr22-38_28785274–29,006,793.1–miR-4319–GRHL1 axis from the CD8^+^ T cell-related ceRNA network were identified as candidates for further analyses. In a survival analysis of CD4^+^ T cell-related DEGs, ADAD1 and DLG3 were strongly correlated with prognosis. Furthermore, 175 small chemical molecule–gene interaction pairs in CD4^+^ T cells and 9 in CD8^+^ T cells were screened. Our identification of T cell-related RNAs, ceRNA network construction, and miRNA target prediction may provide a basis for further studies of colon cancer-induced T cell-mediated immune escape. The prediction of small chemical molecule drugs and survival differences based on differential RNA expression may provide a novel direction for clinical decision-making with respect to treatment and the evaluation of prognosis in colon cancer from the perspective of immunity.

## Supplementary information

**Additional file 1 Table S1**. DEGs between high stromal score vs low stromal score and high immune score vs low immune score.

## Data Availability

The GDC TCGA Colon Cancer (COAD) dataset (https://xenabrowser.net/) and Comparative Toxicogenomics Database (http://ctdbase.org/) are available by contacting the author.

## Abbreviations

MHC-II: Major histocompatibility antigen class II
CTL: CTL
ceRNA: ceRNA
GDC: Genomic Data Commons
TCGA: The Cancer Genome Atlas
KEGG: Kyoto Encyclopedia of Genes and Genomes
GO: Gene ontology
PPI: Protein-protein interaction
ESTIMATE: Estimation of STromal and Immune cells in MAlignant Tumor tissues using Expression data
CTD: Comparative Toxicogenomics Database
OS: Overall surviva
K-M: Kaplan–Meier
ADAD1: Adenosine deaminase domain containing 1
DLG3: Discs Large MAGUK scaffold protein 3
ETS: E26 transformation-specific
mTOR: mammalian target of rapamycin
PLA1: Phospholipase A1
LPI: Lysophosphatidylinositol
GPR-55: G protein-coupled receptor 5
TGFBR2: Transforming growth factor-b receptor 2
GRHL1: Grainyhead-like 1
