# Supplementary material for: Immune-related genes in tumor-specific CD4+ and CD8+ T cells in colon cancer
Source: BMC Cancer. 2020 Jun 22;20:585. doi: 10.1186/s12885-020-07075-x (PMC7310260; doi:10.1186/s12885-020-07075-x)
Supplement: Supplementary file 1 — Additional file 1 Table S1. DEGs between high stromal score vs low stromal score and high immune score vs low immune score. [file 12885_2020_7075_MOESM1_ESM.doc]

| **Supplymentary Table 1. DEGs between high stromal score vs low stromal score and high immune score vs low immune score** | | | | | | | |
| --- | --- | --- | --- | --- | --- | --- | --- |
|  | Stromal | | immune | | Overlapped DEGs | | |
|  | count | Gene names | Count | Gene names | Count | | Gene names |
| Up-regulated DEGs | 5 | | POTEH | | --- | | ZNF354B | | S100A13 | | TSPYL6 | | TOR3A | | 807 | | POTEH | | --- | | ZNF354B | | S100A13 | | TSPYL6 | | TOR3A | | LTB4R2 | | MTX2 | | CWC15 | | CFAP36 | | SLC46A2 | | CRYGA | | HECTD2 | | TANK | | UGT2B15 | | CYP26A1 | | ARMC1 | | SLC25A12 | | FAM209B | | TCN2 | | ZCCHC3 | | AGAP1 | | ACCSL | | EDEM1 | | SYDE1 | | KLHL3 | | OR2M5 | | KLHL9 | | EP400 | | MBTPS2 | | DMWD | | NUPL2 | | SSH3 | | LGMN | | ELF3 | | BPI | | MFSD6 | | RIT2 | | RDH13 | | EPB41L4B | | RSRC1 | | PHYHIPL | | NBPF15 | | TNFSF9 | | FBN2 | | KDELR3 | | RAPGEF3 | | WDTC1 | | CPSF4L | | TBC1D20 | | DENND6A | | RNF217 | | PRLHR | | FCGR1B | | PLK5 | | OSER1 | | PITPNB | | EIF3B | | CERS6 | | SYT1 | | C5orf24 | | CDNF | | MTRR | | MUSK | | TRPA1 | | TAS2R14 | | DIS3 | | DUSP15 | | GOLT1A | | PI16 | | ZSWIM5 | | NR2F1 | | MED24 | | TSKU | | PIP4K2B | | ASCL5 | | NHLRC2 | | THRAP3 | | VNN2 | | SSX2 | | NRARP | | GLMN | | SDF4 | | TIMM8B | | ELMO1 | | SDC1 | | PRRG4 | | C1QTNF6 | | SLC27A3 | | GJC1 | | OR6Y1 | | DLGAP5 | | FTHL17 | | AP1M1 | | PVALB | | MBP | | GLA | | ARL11 | | ORM2 | | EFR3B | | GC | | OR13C7 | | C11orf98 | | DHX33 | | OR52D1 | | DMRT1 | | KIAA2026 | | CDK16 | | TC2N | | RNF7 | | SIRT6 | | CAPN10 | | COMMD6 | | PRSS54 | | KIAA1841 | | ZSWIM1 | | SUPT16H | | MARVELD2 | | REC8 | | RCOR2 | | KMT2E | | OR13D1 | | CD6 | | CD69 | | TIMM8A | | NKAIN4 | | TCEB3C | | RAB11A | | MAFK | | RAB30 | | DUOXA1 | | RAD17 | | DENND5B | | FAM159A | | TXNDC5 | | KCNMB1 | | CALML6 | | SLC17A2 | | CLDN4 | | PPP1R26 | | OTC | | FRG2 | | C1orf159 | | CD79B | | GPATCH3 | | GPR141 | | WDR27 | | SAA1 | | OR9Q2 | | DDA1 | | FOXJ1 | | SOS2 | | GADD45G | | DNAJA4 | | NECAB3 | | ZGRF1 | | PNKD | | GUCY2C | | DUSP8 | | TRIP4 | | AQP11 | | SLC2A12 | | SLC22A25 | | PILRA | | GNA14 | | MYZAP | | RIOK1 | | NSD1 | | ACTR10 | | DTNBP1 | | TFDP3 | | GLRB | | CREB3L4 | | PHF14 | | POLR3A | | AMMECR1 | | RNASE7 | | MEIS2 | | ZNF470 | | SERPINA7 | | SLC6A18 | | CLPTM1 | | SMCO4 | | SDCCAG8 | | CASQ2 | | KLC1 | | ELMOD2 | | ZDHHC23 | | PKD1L1 | | CABLES2 | | UBXN1 | | C3orf62 | | ACCS | | GOLGA8B | | COX7A1 | | OR5V1 | | STX17 | | CWH43 | | OR52N2 | | ZNF79 | | SOSTDC1 | | MBD3L3 | | BCAS2 | | PLA2G4D | | LONP1 | | SDR42E2 | | PLIN5 | | IPO4 | | PYGL | | ZNF585A | | COX7B | | RAD9B | | CHPF2 | | FNTA | | CXCL17 | | MAML2 | | INAFM2 | | CD300C | | PREPL | | CYP20A1 | | THSD7B | | PRIM1 | | DR1 | | TGM5 | | MARK1 | | C16orf92 | | LRP6 | | TMTC1 | | OR8K1 | | ANKRD22 | | TRMT112 | | JUN | | VIMP | | TLR6 | | TM4SF5 | | PPP1R8 | | PALMD | | SCN2A | | SCN9A | | CIDEA | | RPS6KA5 | | ABCB1 | | INADL | | GNB1L | | FANCD2OS | | ERICH3 | | IFIH1 | | ZNF705D | | NOX3 | | ZNF576 | | GLS | | CR1L | | NIP7 | | ATP8B4 | | HCK | | LDHB | | HUWE1 | | GOT1L1 | | PIGH | | PRLH | | RGS13 | | NDUFB3 | | ANKS3 | | LYPLAL1 | | SPPL2C | | PDK2 | | SPINK2 | | HIGD2A | | MPP4 | | IL1RAPL2 | | MAPK7 | | KIAA1429 | | DDX39B | | UBLCP1 | | ATL3 | | CLDN7 | | RRN3 | | CDCA3 | | HOXC11 | | SPSB4 | | DCK | | ETV2 | | GREM1 | | FCF1 | | USF1 | | HNRNPCL3 | | KHDC1 | | FGF5 | | C4orf27 | | CFH | | TRPC1 | | ITPKC | | KRT10 | | UST | | RGS19 | | ABHD17C | | TNNT2 | | MASP1 | | CEP170 | | BDKRB2 | | FPR1 | | SEMA3D | | ACSS1 | | CTRC | | PDZD11 | | EOGT | | STAMBP | | FBXO16 | | PHACTR1 | | PAQR4 | | CCDC67 | | CYP4B1 | | FAM227A | | EPHA1 | | GABRG1 | | SH3TC1 | | RBP5 | | SUSD2 | | PTCH2 | | MYADM | | MS4A6E | | ERN2 | | VAMP5 | | EIF2AK4 | | AQP2 | | SUPT7L | | SUPT20H | | OBP2A | | NCKAP5 | | FAM168A | | KCTD13 | | KLHL6 | | SMYD1 | | DAP3 | | SNX18 | | OR12D3 | | GRIK5 | | RPL6 | | RCHY1 | | MEA1 | | RPL35 | | KLHL10 | | KCNK6 | | PRRX2 | | ZNF257 | | UTP14A | | ZNF260 | | FAM186B | | GATSL3 | | BCAN | | RFX3 | | MZB1 | | TMEM100 | | CLIC3 | | PDE6B | | KCTD1 | | CORIN | | MED15 | | GOLGA8N | | OR6B2 | | XPO7 | | EHD1 | | OR2T2 | | GDA | | BRI3 | | PTPN18 | | HIST1H2AB | | ERICH6B | | HSCB | | CPTP | | GDAP1L1 | | PAK7 | | COQ4 | | GPR1 | | FAHD1 | | C1orf74 | | SF1 | | GNRH1 | | C6orf118 | | LAT | | IFNA5 | | PSG5 | | COX15 | | PGAM2 | | ARCN1 | | SUPT6H | | SOX11 | | WDR78 | | IFNA8 | | FABP5 | | TSPYL2 | | PHKG2 | | PSMC6 | | TSPAN11 | | KANK3 | | PFDN2 | | DES | | PRPF8 | | ONECUT3 | | ABRACL | | DPYSL5 | | ABCA9 | | GDF7 | | MS4A10 | | USP20 | | UCKL1 | | LOXL1 | | LSR | | FAM193B | | PSAPL1 | | RBBP8NL | | C17orf105 | | PROK1 | | GSC | | PCDH11X | | GJB2 | | KLRK1 | | LRCH1 | | CMTM3 | | EXO5 | | YTHDC2 | | PRDM6 | | ZFAND1 | | MYPN | | AK4 | | SPINK9 | | FAM105A | | FGF6 | | TSC22D2 | | HTRA1 | | GALR3 | | MCM6 | | HOMER1 | | VSTM2L | | WRAP53 | | NRROS | | SLC25A11 | | ERC2 | | CSE1L | | SVOPL | | ABCB8 | | LLGL1 | | TBXA2R | | C6orf201 | | ZNF717 | | MYSM1 | | UBE3B | | MALT1 | | KCTD10 | | RANBP10 | | TNFRSF10C | | AIF1L | | ELOVL6 | | SLC25A39 | | ADH1C | | GLUD2 | | FGD6 | | SLC35F3 | | MTAP | | KRTAP9-2 | | AARS | | POLR3H | | C9orf43 | | UMAD1 | | MTMR1 | | C15orf59 | | INS | | C11orf58 | | SNAP91 | | NPY4R | | ALPK2 | | NFRKB | | AIMP2 | | MLF2 | | CXorf40A | | SUFU | | TAAR5 | | ITIH6 | | ZNF740 | | ACTR1B | | PAFAH1B3 | | PRMT8 | | MPP6 | | C9orf57 | | DAPP1 | | SCOC | | ZNF580 | | KRTAP19-1 | | SLC22A5 | | ZNF131 | | PRRT2 | | FAM71C | | HMGN2 | | HIST3H2A | | DFNA5 | | APOPT1 | | RTN1 | | NCAPH2 | | TRIM64C | | GNPDA2 | | KIAA0319L | | PRR14L | | MTMR11 | | NFKBID | | CCDC27 | | UROS | | LIN9 | | NAPSA | | KRT9 | | PDZD4 | | RRBP1 | | GLCCI1 | | NPIPB15 | | APOO | | PFN3 | | SLC12A5 | | TAF12 | | GREM2 | | POLE2 | | TSKS | | GLT8D2 | | TRPV6 | | DNAAF2 | | SMIM20 | | GTF2H2 | | DET1 | | CCDC3 | | CSNK2A1 | | PCBD1 | | FAM181B | | THSD4 | | SPNS3 | | ZNF579 | | TMEM45A | | CES4A | | GLUD1 | | BIN2 | | SCRT2 | | CHPF | | OLIG1 | | TTI1 | | ABR | | RGS10 | | KRTAP20-2 | | MTFMT | | CARS | | SHOX2 | | CKAP5 | | ZBED8 | | GMPPA | | LGALS4 | | ADCK3 | | OCLN | | POU1F1 | | SGPP2 | | ISOC1 | | LIPJ | | GRB10 | | USP17L19 | | CYP4X1 | | CANT1 | | MFSD2B | | NEGR1 | | MX1 | | GCG | | SLC18A2 | | SMC2 | | CYP2C8 | | L3MBTL3 | | CEBPZ | | OR2L3 | | CLEC1A | | ACSL1 | | FAM122A | | RPS15 | | POLK | | XRN2 | | MAP10 | | MARK4 | | ZNF846 | | RTBDN | | TEX38 | | SHH | | TUBA1C | | KCNQ5 | | NAGS | | BCL2L10 | | LAPTM4A | | MAN1C1 | | VBP1 | | COG5 | | PIWIL2 | | C2CD5 | | MORC2 | | TACR3 | | CLEC7A | | RPS6KB1 | | MYADML2 | | NUP188 | | DOCK9 | | ENY2 | | KIR3DL3 | | PCNT | | ARHGEF2 | | KRTAP13-3 | | MRC2 | | IFNL4 | | GRIA2 | | TCEB1 | | CTTNBP2 | | WDPCP | | GPD1L | | T | | FBXL7 | | IFITM3 | | SLITRK1 | | MCOLN3 | | KRT4 | | RIPPLY3 | | CNBP | | CHML | | HOMEZ | | C20orf196 | | DESI1 | | TMEM167A | | ETV3 | | ZNF768 | | SGCE | | FAM188A | | FREM3 | | LEFTY1 | | PLXNA2 | | CAPN5 | | FAF2 | | CAPN8 | | NT5DC3 | | GDE1 | | ORC4 | | CPXCR1 | | SERPING1 | | DUSP10 | | MUC5B | | EFHB | | MRTO4 | | HNRNPCL4 | | BBS9 | | EFCC1 | | CCDC28B | | VDAC1 | | SLC22A24 | | TIPIN | | AXIN2 | | GPR45 | | PMAIP1 | | MTL5 | | IGFL3 | | GSTZ1 | | KIF21A | | ZNF428 | | CNOT11 | | GNAZ | | RTN2 | | SPATA45 | | OR5T3 | | C11orf65 | | F3 | | ZNF18 | | FAM177A1 | | SRP9 | | NXT1 | | SPHK2 | | C11orf91 | | ZNF681 | | GAB1 | | MITF | | PSMD2 | | KCTD18 | | PEPD | | GMNN | | CNNM4 | | SLC9B1 | | RBM20 | | SH3RF1 | | NOD1 | | STXBP3 | | LZTS1 | | C5orf46 | | PDCD10 | | CSMD3 | | WFDC8 | | ASB4 | | SMIM15 | | ASCC1 | | CTDSP2 | | UROC1 | | TMEM139 | | ABI3 | | SAMD4B | | FSIP1 | | CHCHD10 | | DENND1A | | FIBIN | | ULK3 | | MGEA5 | | ZNF696 | | OPLAH | | DAO | | ATP12A | | ADAM9 | | DMKN | | STAT5A | | DNAJB1 | | GOLIM4 | | CORT | | IKBKAP | | PF4 | | C22orf23 | | SPINK13 | | HSPA2 | | NCEH1 | | RP1L1 | | THAP8 | | ZBED6CL | | RASA4 | | SSRP1 | | LRPAP1 | | TAS2R42 | | MPHOSPH10 | | MTRNR2L1 | | BIRC3 | | HDAC7 | | C11orf84 | | PIWIL3 | | GTF3C1 | | DTL | | HIST2H2AB | | TXLNA | | POLD2 | | C14orf80 | | ZNF483 | | HLF | | HBD | | ACSM1 | | PDGFRL | | OR52M1 | | CTDNEP1 | | TRIM41 | | SEC24A | | RAC2 | | WNT5B | | OR14I1 | | OBFC1 | | HTR2B | | MTPAP | | IQCB1 | | IFNK | | KATNA1 | | GEMIN5 | | ALOX12 | | MUS81 | | ATP5D | | ARSD | | UQCRC2 | | GPR153 | | KRTAP3-2 | | NAB2 | | CLEC18A | | TP53BP2 | | KLHL25 | | TNFSF10 | | ZNF781 | | EDN1 | | CRTAP | | TAC3 | | EXOC3 | | ZER1 | | LRRC3B | | S100B | | TIGD2 | | CHGB | | SLC35F2 | | CCT7 | | MAP4K1 | | BRMS1 | | RSPH3 | | RSU1 | | RQCD1 | | ANKRD1 | | SHFM1 | | HDAC4 | | FAM180B | | SLC22A15 | | PDE11A | | DCHS2 | | CORO6 | | UPK3B | | UNC45B | | DGKA | | OLAH | | LILRA2 | | C20orf195 | | UBE2O | | NEFM | | RNF6 | | NPNT | | MRPL17 | | CUTA | | TTC39B | | 5 | | POTEH | | --- | | ZNF354B | | S100A13 | | TSPYL6 | | TOR3A | | |
| Down-regulated DEGs | 78 | | BAX | | --- | | USP50 | | EAF2 | | ANP32D | | ULK2 | | IPP | | ADRB3 | | AHDC1 | | MAGEB5 | | DDHD1 | | PINLYP | | CNOT3 | | TSPAN13 | | ZNF92 | | BEST3 | | GRHL1 | | BRINP3 | | PPFIA3 | | PRRC2B | | GCM2 | | KLHDC7A | | FARSA | | CNGA4 | | SLC37A2 | | CD300E | | POU4F3 | | KDM1B | | SLC22A14 | | COL14A1 | | MKI67 | | BPIFB2 | | SECISBP2L | | C10orf54 | | WHAMM | | ZDHHC5 | | RBL2 | | FAM114A1 | | MRPS26 | | ADD2 | | PSMA8 | | AP4M1 | | ADAD1 | | PLSCR1 | | CD226 | | ITPA | | APOBEC3G | | PYROXD2 | | PLEKHA2 | | RFPL2 | | AADACL3 | | C14orf79 | | CCDC103 | | RUFY1 | | TP53TG3D | | CDH7 | | TAAR1 | | GGCX | | CYB5R4 | | RAB1B | | MCF2L2 | | ZZZ3 | | MISP | | JUND | | GOLGA6A | | DLG3 | | KCNK10 | | HSPA4 | | SCRT1 | | ATOH8 | | KCNK13 | | ELK3 | | IST1 | | PRF1 | | SCGB1D4 | | ECM2 | | RSBN1 | | OR2H2 | | FAM26E | | 463 | | BAX | | --- | | USP50 | | EAF2 | | ANP32D | | ULK2 | | IPP | | ADRB3 | | AHDC1 | | MAGEB5 | | DDHD1 | | PINLYP | | CNOT3 | | TSPAN13 | | ZNF92 | | BEST3 | | GRHL1 | | BRINP3 | | PPFIA3 | | PRRC2B | | GCM2 | | KLHDC7A | | FARSA | | CNGA4 | | SLC37A2 | | CD300E | | POU4F3 | | KDM1B | | SLC22A14 | | COL14A1 | | MKI67 | | BPIFB2 | | SECISBP2L | | C10orf54 | | WHAMM | | ZDHHC5 | | RBL2 | | FAM114A1 | | MRPS26 | | ADD2 | | PSMA8 | | AP4M1 | | ADAD1 | | PLSCR1 | | CD226 | | ITPA | | APOBEC3G | | PYROXD2 | | PLEKHA2 | | RFPL2 | | AADACL3 | | C14orf79 | | CCDC103 | | RUFY1 | | TP53TG3D | | CDH7 | | TAAR1 | | GGCX | | CYB5R4 | | RAB1B | | MCF2L2 | | ZZZ3 | | MISP | | JUND | | GOLGA6A | | DLG3 | | KCNK10 | | HSPA4 | | SCRT1 | | ATOH8 | | KCNK13 | | ELK3 | | IST1 | | PRF1 | | SCGB1D4 | | TMEM119 | | ENTPD6 | | HSD17B14 | | MRPL47 | | C11orf85 | | RAP1GAP2 | | THOP1 | | TMEM115 | | RFC1 | | MIA | | FNIP2 | | ANKRD46 | | HHIPL2 | | SUCNR1 | | KLKB1 | | PIWIL4 | | LMO7 | | MTERF1 | | ZNF75A | | DCAF12L1 | | ELAVL3 | | ARR3 | | TCP10 | | IMPA2 | | UBE4B | | STEAP1B | | IL2RB | | ZNF37A | | PTTG1IP | | SYT9 | | PLEKHF2 | | MC2R | | KIF26B | | SENP8 | | POGK | | SLC44A4 | | PRDM2 | | PTGER3 | | GPR42 | | ITPRIP | | CYP17A1 | | VLDLR | | IKZF2 | | CDC27 | | MYH10 | | MUC13 | | HAS1 | | TOLLIP | | F13B | | AKAP8 | | SLC38A2 | | PPM1J | | FILIP1 | | HIST2H3A | | TCEAL3 | | TCIRG1 | | OR5T2 | | DIDO1 | | REC114 | | AGL | | PELI3 | | C1QL1 | | P4HTM | | MDFI | | TMEM178A | | PRAMEF19 | | PMS2 | | SERINC5 | | CARTPT | | CXCR3 | | DLX5 | | HES7 | | CSNK1A1L | | C7orf34 | | IGLL5 | | C8orf33 | | MEN1 | | GABBR1 | | RXFP2 | | HEXDC | | ECH1 | | MMP10 | | OR1D5 | | TAF1L | | SYNPO2 | | FUS | | ATG4C | | NTHL1 | | OR10G4 | | TMC1 | | PCED1A | | ATP13A4 | | SNRPA | | ASB5 | | MGME1 | | FLOT2 | | OSTN | | CAPN1 | | HEY2 | | MAPK11 | | NUP205 | | GTSE1 | | LURAP1L | | PAX6 | | LY6D | | RPS8 | | HOXA1 | | AGXT | | COL9A2 | | RUNX1 | | RPLP0 | | LHFPL5 | | STRADA | | CCL27 | | RANBP2 | | SOX4 | | TAS1R1 | | PRSS21 | | ATIC | | MSTN | | COQ5 | | NOBOX | | MRPS24 | | TMEM26 | | DOC2A | | ETS1 | | MRPS18A | | ITM2A | | C3 | | RBP3 | | PDAP1 | | TMEM222 | | RALB | | COG2 | | OR2V1 | | ZNF555 | | TRIT1 | | NPY2R | | GATA6 | | PDE6H | | ANG | | ZNF560 | | CFAP54 | | CTSG | | SLC35B3 | | ESCO2 | | ATF7IP | | CHD1L | | PRKCD | | DDX3Y | | COX5A | | NHP2 | | GSTO1 | | RAD23A | | C2orf72 | | HIVEP1 | | ARL6IP6 | | CUTC | | UXS1 | | OPRK1 | | LRFN5 | | TIMM23B | | INO80B | | INIP | | CHST12 | | ERICH6 | | ST3GAL2 | | AIP | | AIFM3 | | ANKDD1B | | DNAJC28 | | PRPF4 | | COL9A3 | | SNAI2 | | SRR | | OST4 | | DRC7 | | BNC2 | | NFASC | | DDTL | | MRPL42 | | DNASE1L3 | | MTURN | | TRIM58 | | C1orf116 | | PIK3CA | | DMRTC1 | | POU4F2 | | TRAPPC4 | | MCHR2 | | MRM1 | | SNRPC | | KIF26A | | C2orf69 | | RPS12 | | GLT6D1 | | UBE2Q2 | | BIRC8 | | UVRAG | | MYEOV2 | | ATP6V0C | | HEATR5B | | ZNF367 | | OSBP | | C5orf49 | | MAGEF1 | | CAMKV | | PAQR7 | | VPRBP | | PHLDB2 | | ELK1 | | B4GALT5 | | LRRK2 | | ACPT | | CSNK1G1 | | DPYSL3 | | GAGE13 | | MUSTN1 | | ITGBL1 | | CTAG1B | | PLCB2 | | TIMMDC1 | | AKAP5 | | NLRC5 | | SLC2A5 | | C19orf47 | | KDM4D | | WDYHV1 | | TTC22 | | SUGP1 | | BICD2 | | MOS | | PPP1R3G | | ZNF284 | | DECR1 | | NKX3-2 | | PRELID1 | | TECR | | KCNH6 | | IGSF11 | | ZFP92 | | TSPAN14 | | OR7A10 | | KRTAP4-2 | | PUM1 | | USP7 | | BOLA3 | | RPS20 | | BRF1 | | CYLD | | BIRC7 | | RAVER1 | | ENTHD1 | | GPAA1 | | LRRC41 | | TM6SF1 | | PHF11 | | SERPINB13 | | FBXO4 | | NOP56 | | GPR108 | | MRPL13 | | RBMXL3 | | KBTBD3 | | ERCC5 | | WBP4 | | ZAR1 | | SLC25A17 | | TBCE | | SH2D1B | | SMU1 | | NUCKS1 | | TMEM102 | | KIAA1161 | | DCTN1 | | RFNG | | ESYT1 | | YIF1B | | KIAA1586 | | MS4A7 | | RPL36 | | HOXD12 | | MLC1 | | EEF1B2 | | CLPX | | OR4A16 | | LBX1 | | MTRF1 | | PRAMEF1 | | NBPF9 | | ZFYVE26 | | PRR20E | | CDKN1C | | PRRC1 | | ACAD11 | | PCDH12 | | FBLN1 | | GCNT2 | | PRKCA | | SLC6A3 | | CHIT1 | | ZNF319 | | ZNF600 | | SUPT3H | | TUBGCP5 | | ZNF852 | | POM121C | | KDELC1 | | WDFY3 | | MAGEB16 | | KRT222 | | MT1HL1 | | LCE3E | | NIM1K | | SLC12A6 | | SPAG16 | | PKD2L2 | | LGALS3BP | | ADRA1D | | UBA1 | | VPS45 | | OR51B5 | | FBLIM1 | | GJD3 | | GALT | | KCNQ2 | | TIFA | | BHLHE40 | | VWC2 | | KCNC4 | | ZNF697 | | WDR3 | | TRIM49B | | POLR3B | | KIF9 | | FAM180A | | FCER1G | | CNN1 | | SMKR1 | | TMEM217 | | UBASH3A | | SDHC | | TBX15 | | CDH23 | | PIP5K1A | | DSC3 | | RNASE2 | | SLC38A3 | | ZNF593 | | ZSCAN23 | | EIF1AD | | ETHE1 | | SGK2 | | CCDC96 | | LIM2 | | DCN | | SLC16A12 | | OTUD4 | | FANCL | | ARC | | YY2 | | ARHGAP36 | | PLXNA1 | | LANCL3 | | BTN2A1 | | GPR82 | | LUM | | SEC61B | | CWC22 | | A4GNT | | BBS2 | | HOXD9 | | MAPK12 | | LY6E | | LYPD4 | | XPC | | MEX3A | | TCEAL1 | | HAPLN2 | | N6AMT1 | | NPRL3 | | SYNC | | R3HDM4 | | DGKB | | CLTB | | IL36B | | ST3GAL1 | | NDUFAB1 | | HIST2H3D | | 74 | | BAX | | --- | | USP50 | | EAF2 | | ANP32D | | ULK2 | | IPP | | ADRB3 | | AHDC1 | | MAGEB5 | | DDHD1 | | PINLYP | | CNOT3 | | TSPAN13 | | ZNF92 | | BEST3 | | GRHL1 | | BRINP3 | | PPFIA3 | | PRRC2B | | GCM2 | | KLHDC7A | | FARSA | | CNGA4 | | SLC37A2 | | CD300E | | POU4F3 | | KDM1B | | SLC22A14 | | COL14A1 | | MKI67 | | BPIFB2 | | SECISBP2L | | C10orf54 | | WHAMM | | ZDHHC5 | | RBL2 | | FAM114A1 | | MRPS26 | | ADD2 | | PSMA8 | | AP4M1 | | ADAD1 | | PLSCR1 | | CD226 | | ITPA | | APOBEC3G | | PYROXD2 | | PLEKHA2 | | RFPL2 | | AADACL3 | | C14orf79 | | CCDC103 | | RUFY1 | | TP53TG3D | | CDH7 | | TAAR1 | | GGCX | | CYB5R4 | | RAB1B | | MCF2L2 | | ZZZ3 | | MISP | | JUND | | GOLGA6A | | DLG3 | | KCNK10 | | HSPA4 | | SCRT1 | | ATOH8 | | KCNK13 | | ELK3 | | IST1 | | PRF1 | | SCGB1D4 | | |

DEGs: differentially expressed genes.
